# *Bifidobacterium breve* UCC2003 Exopolysaccharide Modulates the Early Life Microbiota by Acting as a Potential Dietary Substrate

**DOI:** 10.3390/nu12040948

**Published:** 2020-03-29

**Authors:** Deborah Püngel, Agatha Treveil, Matthew J Dalby, Shabhonam Caim, Ian J Colquhoun, Catherine Booth, Jennifer Ketskemety, Tamas Korcsmaros, Douwe van Sinderen, Melissa AE Lawson, Lindsay J Hall

**Affiliations:** 1 Gut Microbes & Health Institute Strategic Programme, Quadram Institute Bioscience, Norwich NR4 7UQ, UK; deborah.pungel@tum.de (D.P.); agatha.treveil@earlham.ac.uk (A.T.); Matthew.Dalby@quadram.ac.uk (M.J.D.); shabhonam.caim@cegx.co.uk (S.C.); Jennifer.KETSKEMETY@mpbio.com (J.K.); tamas.korcsmaros@earlham.ac.uk (T.K.); 2 Earlham Institute, Norwich NR4 7UZ, UK; 3 Analytical Sciences, Quadram Institute Bioscience, Norwich NR4 7UQ, UK; ianjcolquhoun@gmail.com (I.J.C.); Catherine.Booth@quadram.ac.uk (C.B.); 4 APC Microbiome Institute, University College Cork, T12 K8AF Cork, Ireland; d.vansinderen@ucc.ie; 5 Lydia Becker Institute for Immunology and Inflammation, Wellcome Trust Centre for Cell Matrix Research, Division of Infection, Immunity and Respiratory Medicine, School of Biological Sciences, Faculty of Biology, Medicine and Health, University of Manchester, Manchester Academic Health Science Centre, Manchester M13 9PL, UK; 6 Norwich Medical School, University of East Anglia, Norwich NR4 7TJ, UK

**Keywords:** *Bifidobacterium*, exopolysaccharides, early life, diet, model colon, cross-feeding, 16S rRNA profiling, metabolomics

## Abstract

Background: *Bifidobacterium* represents an important early life microbiota member. Specific bifidobacterial components, exopolysaccharides (EPS), positively modulate host responses, with purified EPS also suggested to impact microbe–microbe interactions by acting as a nutrient substrate. Thus, we determined the longitudinal effects of bifidobacterial EPS on microbial communities and metabolite profiles using an infant model colon system. Methods: Differential gene expression and growth characteristics were determined for each strain; *Bifidobacterium breve* UCC2003 and corresponding isogenic EPS-deletion mutant (*B. breve* UCC2003del). Model colon vessels were inoculated with *B. breve* and microbiome dynamics monitored using 16S rRNA sequencing and metabolomics (NMR). Results: Transcriptomics of EPS mutant vs. *B. breve* UCC2003 highlighted discrete differential gene expression (e.g., *eps* biosynthetic cluster), though overall growth dynamics between strains were unaffected. The EPS-positive vessel had significant shifts in microbiome and metabolite profiles until study end (405 h); with increases of *Tyzzerella* and *Faecalibacterium*, and short-chain fatty acids, with further correlations between taxa and metabolites which were not observed within the EPS-negative vessel. Conclusions: These data indicate that *B. breve* UCC2003 EPS is potentially metabolized by infant microbiota members, leading to differential microbial metabolism and altered metabolite by-products. Overall, these findings may allow development of EPS-specific strategies to promote infant health.

## 1. Introduction

Members from the genus *Bifidobacterium* represent one of the dominant bacterial groups in the early life gut microbiota, with high levels associated with improved infant health [1,2,3,4,5]. Unlike the adult gut microbiome, the infant microbiome is less stable, and dietary change is proposed to lead to severe shifts in the abundance of major bacterial taxa over time [3,6,7]. The gut microbiota of breast-fed infants is dominated by *Bifidobacterium* (approx. 80% of the total community), which represents an important microbial pioneer or founder genus [8,9]. In contrast, formula-fed infants have a more diverse microbiota and *Bifidobacterium* comprise a smaller proportion (approx. 5%–30%) [10]. The introduction of solid food at weaning marks a transition into a more complex microbiome, with a concurrent reduction in *Bifidobacterium* levels, likely due to the loss of milk as a sole dietary source. Notably, during these phases of significant dietary change, there is a shift in bifidobacterial species and strains, which may link to the wider repertoire of enzymes capable of digesting a more ‘adult’ diet [2,4,6,11].

Currently, only a small number of studies have explored the mechanisms by which *Bifidobacterium* species modulate the wider microbiota and/or provide benefits to the host. Several groups have provided data indicating that many *Bifidobacterium* species and strains produce exopolysaccharides (EPSs), which appear to have a variety of roles in microbe–host and microbe–microbe interactions [12]. Bacterial EPS are polymerized mono- or oligo-saccharides which form a diverse range of homo- or hetero-polysaccharides, that can be linked to the bacterial cell wall or secreted. Analyses of several *Bifidobacterium* species has revealed the presence of EPS gene clusters [13], with chemical analysis indicating glucose and galactose as major components, with very low levels of rhamnose also present throughout these structures [14,15]. To date, most studies have focused on EPS-host interactions including: adhesion to host cells, and modulation of epithelial and immune responses [16,17,18]. 

Microbe–microbe interactions are a key feature of community structuring and are often mediated through cross-feeding of microbial-derived metabolites [19]. Interestingly, bifidobacterial EPS has previously been reported to act as a nutrient source for other bacteria within microbial communities. In two independent studies, Salazar and colleagues found that isolated EPS from human-associated *Bifidobacterium* species may act as a fermentable substrate for other members of the microbiota in faecal batch cultures (which was species and strain dependent) [20,21]. More recently, this inter-microbial cross-feeding was also shown *in vitro,* with EPS from *Bifidobacterium animalis* subsp. *lactis* and *Bifidobacterium longum* promoting growth of *Bacteroides fragilis,* which correlated with increased short-chain fatty acid (SCFA) production [22]. 

Collectively, these studies suggest a potential role for EPS in microbe–microbe interactions, by acting as a substrate within gut microbiota cross-feeding networks. However, the exact role of bifidobacterial EPS in community restructuring is currently unclear as previous studies supplemented vessels with purified EPS, rather than administrating an EPS-producing *Bifidobacterium* strain (which would mimic the actual gut environment). Here, we explored the effects of *B. breve* UCC2003 (EPS-positive) and *B. breve* UCC2003del (EPS-negative) on the microbiota using an infant model colon system. We established differential gene expression and growth characteristics *in vitro* of each strain, prior to supplementation, and then performed longitudinal microbiota (via 16S rRNA) and metabolomic (via NMR) profiling, which indicated that EPS produced by *B. breve* UCC2003 may function as a potential dietary substrate that induces remodelling of the early life microbiota. 

## 2. Materials and Methods 

### 2.1. Bacterial Strains and Growth Conditions

The *B. breve* UCC2003 strains used in this experiment included *B. breve* UCC2003 (i.e. EPS-positive, and isolated from infant stool), and EPS deletion mutant *B. breve* UCC2003del (i.e. EPS-negative, [16]). All strains were cultured in reinforced Clostridium medium (RCM) and deMan-Rogosa-Sharpe (MRS) medium and supplemented with 0.05 mg/mL L^-^cysteine (L-cys) HCl anaerobically at 37 °C for upwards of 48 h. When measuring growth kinetics samples were taken every 2 hours from 0 h to 12 h and 24 h to 30 h, with a measurement after 48 h of growth. Optical density (600 nm; OD_600_) and pH values were recorded at each time point. For colony forming units (CFU) per mL determination, samples were plated onto RCM agar plates containing 0.05 mg/mL L-cys-HCl and grown anaerobically for 48 h. 

### 2.2. B. breve Transcriptomics and Bioinformatics Analysis 

To identify potential EPS modulated genes, pre-cultures grown in MRS media overnight (as above) were used to inoculate fresh cultures and cells were harvested at the exponential (approx. 8 h) phase, PBS washed, and RNA extraction was performed immediately. The cells were resuspended in 600 µL RLT lysis buffer containing 8 µL β-mercaptoethanol. The suspension was transferred to Fastprep Lysing Matrix E tube (MP Biomedicals) and was homogenized using FastPrep-24 instrument (MP Biomedicals, Irvine, CA, USA) at 6.0 m/s for 3 × 1 min with 5 min resting intervals on ice. Samples were centrifuged at 14,000 *× g* for 10 min and RNA was extracted using Qiagen RNeasy mini plus kit (Qiagen, Hilden, Germany) according to the manufacturer’s instructions. The RNA quality and concentration were determined using Agilent 2100 Bioanalyzer (Agilent Inc. Santa Clara, CA, USA). Only samples with RIN values above eight were sequenced. Isolated RNA was processed with Ribo-depletion, and samples sequenced on HiSeq V4 75bp (Illumina, Cambridge, UK) using non^-^stranded, paired end reads.

For computational analysis, the quality of stranded reads was assessed by FastQC software (version 0.11.8) [23]. Reads were aligned against the full nucleotide sequence of *B. breve* UCC2003 (RefSeq: NC_020517). The alignment and quantification was performed using Kallisto (version 0.44.0), the quantified read data was normalized and differential expression analysis was conducted using DeSeq2 (version 1.22.2) [24,25]. Genes with an absolute log2 fold change ≥1 and *p* adj value ≤ 0.05 were considered to be differentially expressed. PCA plots, heatplots and plots of differential expression across the genome were generated using custom R scripts. All genes in the *B. breve* UCC2003 genome (RefSeq: NC_020517) were functionally annotated with categories and descriptions using EggNog-mapper (version 4.5) [23]. Custom R scripts were used to test for enrichment of EggNog functional categories in the upregulated and downregulated differentially expressed genes. This analysis was carried out using gene set enrichment functions of the R package clusterProfiler (version 3.10.1) [26]. All raw reads were deposited in European Nucleotide Archive: PRJEB35291. 

### 2.3. Transmission Electron Microscopy (TEM)

Strains were grown as described above, and after 48 h growth, cells were centrifuged at 4500 × *g* at 4 °C for 10min with low break. Bacterial pellets were resuspended in 1mL of fixative comprising 2.5% glutaraldehyde in 0.05 M sodium cacodylate pH 7.2. Fixation was carried out for 1.5 h at room temperature, after which cell suspensions were centrifuged (7.5 xg rpm, 3 min) and washed in 0.05 M Sodium Cacodylate buffer ×3 (10 min). After final centrifugation, cell pellets were mixed 1:1 with molten 2% low gelling temperature agarose, solidified by chilling, and then chopped into 1mm^3^ pieces. Sample pieces were post fixed in 1% OsO_4_ for 2 h followed by washing ×3 (15 min) in deionized water. Sample pieces were then dehydrated through an ethanol series (30%, 50%, 70%, 90%, 100% ×3) for at least 15 min in each ethanol dilution. Samples were infiltrated with a 1:1 mix of LR White medium grade resin to 100% ethanol, followed by a 2:1 and a 3:1 mix and finally 100% resin, with one hour between each change. This was followed by two more changes into fresh 100% resin, with periods of eight hours between. Four blocks/sample were put into Beem capsules (Size 00, EMS, Hatfield, PA, USA) with fresh resin and polymerized for 24 h at 60 °C. Sections approximately 90 nm thick were cut using an ultramicrotome with a glass knife, collected on formvar/carbon coated 200 mesh copper grids, stained sequentially with 2% uranyl acetate for 1 h at room temperature, and added to 0.5% lead citrate-tribasic trihydrate for 1min at room temperature. Deionised water washes were performed (×5) following each of the staining steps. Sections were examined and imaged in a Talos F200C transmission electron microscope (Thermo Fisher, Waltham, MA, USA) at 200 kV with a “Gatan One View” digital Camera. 

### 2.4. Infant Model Colon System

All subjects gave their informed consent for inclusion before they participated in the study. The study was conducted in accordance with the Declaration of Helsinki, and the protocol was approved by the Quadram Institute Bioscience Ethics Committee and in accordance with protocols by the National Research Ethics Service (NRES) approved UEA/QIB Biorepository (Licence no: 11208). Faeces were collected from healthy, full-term breast-fed infants (aged from seven months to one year old). One-gram samples from five frozen stool samples were homogenized with 5mL reduced PBS (500 µL of 3% L-cys HCl in 50mL PBS), filtered (70 µm) and evenly distributed to each vessel. Samples were pooled based on previous work indicating combined samples robustly demonstrated a median value of the donors and provided a more stable environment for downstream microbiome and metabolite analysis [27]. Model colon system media was made according to Cinquin et al., and included a vitamin solution (pantothenate 10 mg/L, nicotinamide 5 mg/L, thiamine 4 mg/L, biotin 2 mg/L, vitamin B12 0.5 mg/L, menadione, 1 mg/L and p-aminobenzoic acid 5 mg/L) as described in Gibson and Wang [28,29]. The continuous-fed batch cultivation was performed in 1.4 L bioreactors (Multifors) and monitored with Eve® software (Infors AG, Basel, Switzerland). Parameters were set according to [28]; briefly, vessels were maintained at 37 °C, pH 6.7 and anaerobic, with a media exchange occurring every 12 h (retention time). All vessels were given 10 days to permit equilibrium of the microbiota to the *in vitro* vessel environment.

For inoculation of *B. breve,* strains were grown in the infant gut media. Overnight cultures were then centrifuged at 4500 *× g* for 10 min at 4 °C, washed with PBS and then inoculated into each vessel. *B. breve* UCC2003 EPS-positive 4.4 × 10^8^ CFU/vessel, and *B. breve* UCC2003del EPS-negative 2.4 × 10^8^ CFU/vessel was added. Samples were snap frozen in liquid nitrogen at each time point t = 0, 6, 12, 24, 36 hours post inoculation with *B. breve*, and once a day from that time point onward. 

### 2.5. DNA Extraction, 16S rRNA Library Preparation, Sequencing, and Bioinformatics Analysis

For genomic DNA extraction of the samples FastDNA® Spin Kit for Soil (MP Biomedicals, Irvine, CA, USA) was used according to the manufacturer’s instructions with an additional two bead-beating runs of 60 s and setting 6.0 m/s. DNA quantification was determined with the Qubit® 2.0 Fluorometer (Thermo Fisher, Waltham, MA, USA) using the broad range Qubit® Assay Kits for DNA, RNA and Protein following manual instructions, and DNA was normalized to a final concentration between 4–20 ng/µL prior to 16S PCR analysis.

V1 and V2 of the 16S rRNA gene were targeted and amplified using a Q5 high-fidelity polymerase kit. Primer sequences for amplification are found in Table S1. PCR amplification conditions were: 1 cycle of 98 °C for 2 min, followed by 20 cycles of 98 °C for 30 s, 50 °C for 30 s and 72 °C for 90 s, and a final cycle of 72 °C for 5 min. PCR products were purified using Ampure XP beads (Agencourt, Beckman Coulter, Brea, CA, USA) and pooled in an equivalent molar mix based on the concentration of each PCR product determined by Quant^-^iT™ PicoGreen® dsDNA Assay Kit (Thermo Fisher, Waltham, MA, USA ). Sequencing was performed on an Illumina MiSeq platform using paired end reads (average read length was 330 bp). 16S rRNA gene sequencing analyses and bioinformatics analyses were performed as previously published [30]. All raw reads were deposited in the European Nucleotide Archive: PRJEB35291.

### 2.6. ^1^H NMR Analyses

For the analysis, 50 mg of model colon samples were mixed with 700 μL NMR buffer (0.26 g NaH₂PO, 1.44 g K_2_HPO_4_, 17 mg TSP, 56.1 mg NaN_3_ and 100 mL D_2_O) and centrifuged at 14000 *× g* at 4 °C for 5 min. Then, 600 μL of supernatants were transferred into NMR tubes (GPE Scientific Ltd, Leighton Buzzard, UK) and the ^1^H NMR spectrum was recorded at 600 MHz on a Bruker Avance spectrometer (Bruker BioSpin GmbH, Rheinstetten, Germany) running Topspin 3.2 software. Each ^1^H NMR spectrum was acquired with 128 scans, a spectral width of 12300 Hz and an acquisition time of 2.7s. The “noesygppr1d” presaturation sequence was used to suppress the residual water signal with a low power selective irradiation at the water frequency during the recycle delay (3 s). Spectra were transformed with a 0.3-Hz line broadening, automatically phased, baseline corrected, and referenced by setting the TSP methyl signal to 0 ppm. Metabolites were identified and subsequently quantified using a library of reference standard spectra provided with the Chenomx NMR suite 8.4™ software (Edmonton, AB, Canada).

### 2.7. Statistical Analyses and Plotting

Statistical analyses on growth curves were performed in GraphPad Prism version 5.04 (San Diego, CA, USA). Mann-Whitney-U-Test was performed for cultures in MRS. Error bars denote standard deviation (SD). All plots were produced using either GraphPad Prism version 5.04 or R Studio version 1.1.463 using the ggplot2 R package version 3.1.0. For NMDS (Non-metric multidimensional scaling) in R Studio, the 16S rRNA bacterial sequence data were subsampled to an even depth of 120,070 sequences using phyloseq package version 1.24.2. NMDS plots were generated with a Bray–Curtis dissimilarity calculation in R Studio used with the vegan package version 2.5-4 in R Studio version 1.1463 with R 3.5.0. The Spearman correlation analysis was calculated using Kendall in R Studio (version 1.1463).

## 3. Results

### 3.1. Surface-Associated EPS Influences B. breve Gene Expression 

**Figure 1:**
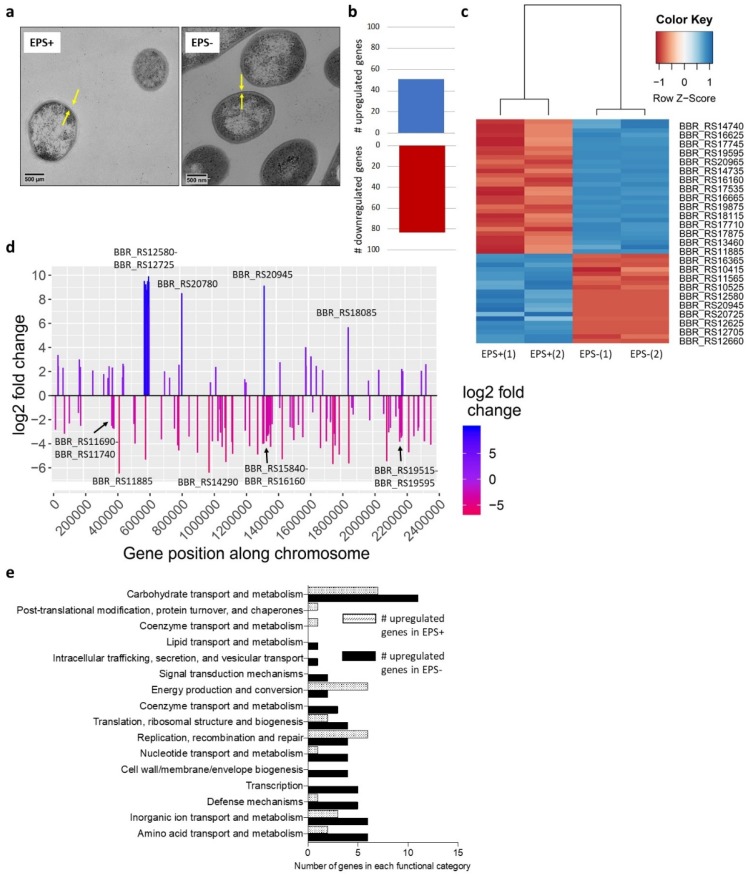
Characterization of EPS-mediated *B. breve* modulation. (**a**) TEM of *B. breve* UCC2003 (EPS+) and *B. breve* UCC2003del (EPS−). Arrows note the EPS layer. (**b**) Total number of differentially expressed genes when comparing EPS+ to EPS− conditions, (two independent experimental repeats). (**c**) Top 50 differently expressed genes with an absolute log2 fold change ≥1 and p adj value ≤ 0.05. Hierarchical clustering of samples. Significance based on adjusted p value. (**d**) Bar plot of EPS+ vs EPS- differential gene expression (absolute log2 fold change ≥1 and p adj value ≤ 0.05) across the genome with gene name labels. (**e**) Number of differentially expressed genes in each functional category (EPS+ vs EPS−) according to EggNog mapper annotation.

Pure bacterial cultures were subjected to TEM to visualize the presence and absence of EPS prior to model colon experiments. Images indicated EPS-positive *B. breve* UCC2003 bacteria had a thicker and differentially stained cell wall (as indicated by arrows, Figure 1a), in contrast to the EPS-negative strain (i.e. *B. breve* UCC2003del) which is in line with previously published data [16]. 

Previous work has suggested that bacterial EPS may act as a signalling molecule, and therefore its absence may alter bacterial gene expression [31]. To examine this, we grew *B. breve* EPS-positive and *B. breve* EPS-negative in culture until mid-exponential phase and isolated mRNA for transcriptome analysis. Principal component analysis (PCA) showed separation between EPS-positive and EPS-negative conditions (45% of variance, Figure S1a); in addition, we found 51 upregulated and 83 downregulated genes (comparing EPS-positive to EPS-negative with an absolute log2 fold change ≥1 and p adjusted value ≤ 0.05; Figure 1b,c). Annotation of regulated genes indicated high differential expression around the *B. breve* EPS cluster (Figure 1d; cluster at position ~600,000). The EPS-related cluster was the only full gene cluster differently regulated between the two isolates during growth in rich media, suggesting that these strains are highly similar despite the lack of EPS structure on the mutant strain. Using the EggNog mapper to functionally classify differentially expressed genes (from EPS+ vs EPS− cultures), the majority of genes were assigned to unknown function (18 genes up-regulated in EPS+ and 25 genes upregulated in EPS−, data not shown). In general, we found that the EPS− strain had a greater number of significant genes upregulated in metabolic pathways (including carbohydrate, nucleotide, inorganic ion, lipid and amino acid transport/metabolism) and bacterial cell growth (cell wall/membrane/envelope biogenesis and transcription; Figure 1e). Overall the EPS+ strain had fewer significantly differentially expressed genes, but in contrast to EPS−, EPS+ bacteria had a higher proportion of gene expression related to replication/recombination and repair, energy production and post-translation modification/protein turnover/chaperones and coenzyme transport and metabolism (Figure 1e). Collectively, this data suggests that the absence of EPS alters gene expression of many metabolic pathways, but importantly it does not influence overall growth kinetics (Figure S1b). 

### 3.2. B. breve EPS Influences Infant Microbiome Composition over Time

**Figure 2:**
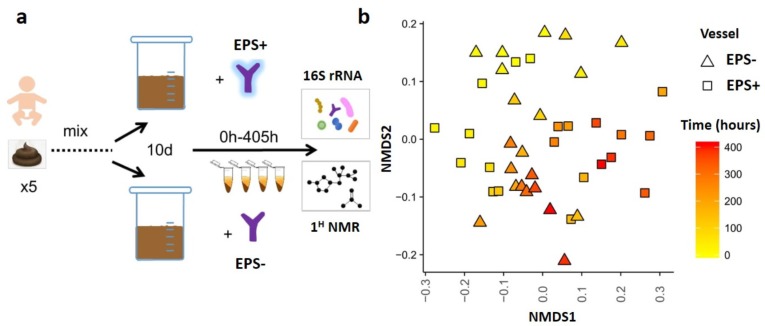
Experimental set-up and NMDS plots of EPS+ and EPS− vessels. (**a**) Five different stool samples from four different infants, age 6–12 months, were combined, processed and added to each Vessel (see Methods). Vessels were acclimatized for ten days, followed by inoculation with, Vessel A: *B. breve* UCC2003 EPS+, and Vessel B: *B. breve* UCC2003del EPS−. Aseptic sampling was performed from 0–408 h after inoculation and processed for 16S rRNA analyses and NMR. Samples were taken before inoculation (t = 0), at time points (hours after inoculation) 6, 12, 24, 36 and from 48 h to 408 h every 24 h. (**b**) NMDS plot using a Bray–Curtis dissimilarity calculation, for both vessels and separately (Figure S2). Changes over time are coloured from yellow (time point 0 h) to red (time point 408 h).

To determine if bifidobacterial EPS acts as an additional nutrient substance, impacting cross-feeding activities, *B. breve* strains were inoculated into a complex infant model colon system (as depicted in Figure 2a). We used 16S rRNA gene analysis to probe kinetics of bacterial community changes induced by the presence of *B. breve* EPS-positive or EPS-negative strains over 405 hours of culture. 

**Figure 3:**
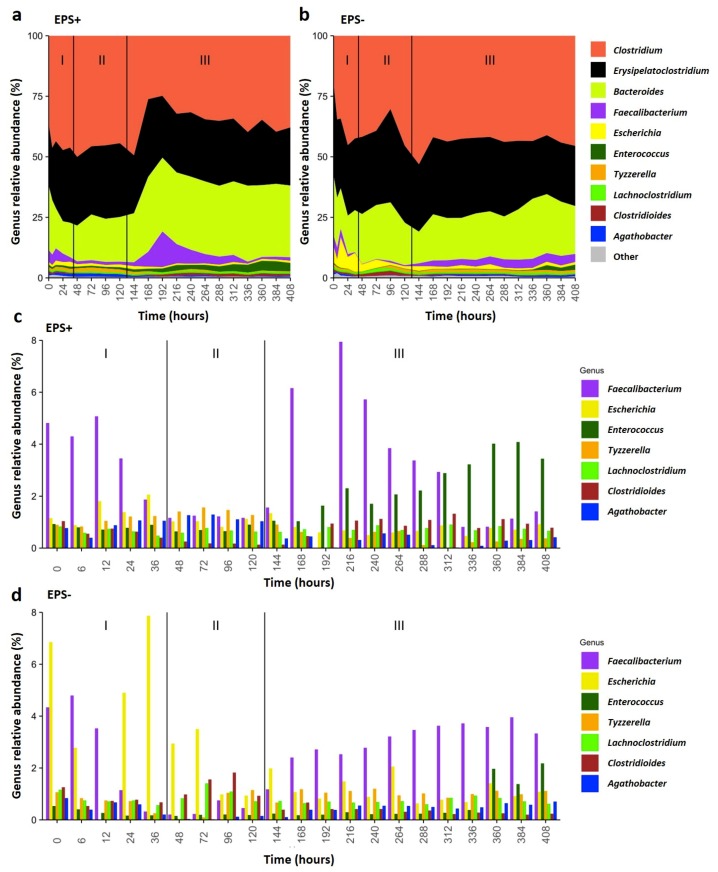
Microbiota profiling in EPS+ and EPS− vessels. Area plot of proportional read counts and total genus abundance of 16S rRNA gene analyses of (**a**) EPS+ and (**b**) EPS− model colon vessels. (**c**) EPS+ and (**d**) EPS− vessel changes (by phase) in the abundant genera.

To offset the variability of microbial community dynamics that occur when stool samples from multiple infants are mixed and grown together in batch culture, all vessels were acclimatized for ten days to stabilize ecosystems prior to *B. breve* supplementation at t = 0 (Figure S2). *B. breve* levels were monitored over time by analysis of species-specific 16S rRNA abundance [30]; this method indicated that *B. breve* reads were only detectable at time 0 h (following inoculation of each vessel) (Figure S4a,b). Sampling at 6 h–36 h post-supplementation showed presence of *B. breve*, suggesting short-term colonization within the infant model colon system. After a full media exchange (approx. 12 h), *B. breve* accounted for approximately half of the total *Bifidobacterium* relative abundance within the EPS-positive vessel, and one-third of the total bifidobacterial abundance in the EPS-negative vessel (Figure S4a,b). This is perhaps unsurprising, since it has been previously reported that once stability has been reached in the microbiota, only one-third of individuals are colonized with *Bifidobacterium* after supplementation [32]. Despite short-term colonization, overtime microbial diversity in each vessel shifted as indicated by NMDS plots of total taxonomic profiles (Figure 2b, Figure S3a,b), with specific changes in microbial composition and abundancy observed in each vessel throughout the experimental period (Figure 3 and Figure S5, Tables S2 and S3). Comparing the wider bacterial genus, community profiles of the vessels indicated that both EPS-positive and EPS-negative conditions had similar core microbiomes; therefore, we chose to focus our analysis on the top five genera in each condition. All vessels were dominated with a high proportion of (1) *Clostridium*, (2) *Bacteroides* and (3) *Erysipelatoclostridium* across all time points (Figure 3a,b). Within the EPS+ vessel *Faecalibacterium* was the fourth most abundance genera across most time points; whilst bacteria belonging to the genera *Escherichia* were the fifth most prevalent at 0–36 h, from 48–129 h *Tyzzerella* was fifth, and *Enterococcus* from 144–408 h (Figure 3a,c and Table S2). These shift in the top five genera within vessels corresponded to three unique microbial ‘phases’ (I, II, and III). Notably, the presence of EPS (i.e. EPS+ vessel) correlated with different ecosystem structuring (Figure 3a,c and Table S2) when compared to EPS– vessel (Figure 3b,d and Table S3). Earlier time-points (0–36 h) showed similar relative genus abundances; *Clostridium*, *Bacteroides*, *Erysipelatoclostridium, and Faecalibacterium*, with *Escherichia* higher in the EPS– vessel. In the second phase from 48–120 h, *Faecalibacterium* relative abundance was decreased in both vessels (although not as significantly in the EPS+ vessel), with proportions of *Clostridioides* increased in the EPS− vessel. In phase I and crossing to phase II, we observed an increase in *Tyzzerella* in the EPS+ vessel (from 12 h). In phase II (48–120 h), *Lachnoclostridium* and *Tyzzerella* had increased read counts, whereas *Escherichia* decreased in the EPS− vessel. In the last phase (III), at 168 h–312 h, we observed a spike in *Faecalibacterium* in the EPS+ vessel, followed by a ‘wave’ of *Enterococcus* (192–408 h), whereas the number of reads from *Tyzzerella* decreased towards the end (Figure 3a, c; purple for *Faecalibacterium*, green for *Enterococcus*). The EPS− vessel was observed to have a steady increase in relative abundance of *Faecalibacterium*, with overall reductions in *Clostridioides* (Figure 3b,d; purple for *Faecalibacterium*, green for *Enterococcus*). These profiles indicate distinct and major changes in bacterial genera in response to *B. breve* UCC2003-associated EPS. 

Using Spearman correlation analysis, we next assessed if the presence of total *Bifidobacterium* correlated with other bacterial genera (Figure S6). Overall, we noted that most genera were found to be (weakly) negatively correlated in the EPS+ vs. the EPS– vessel, with a strongly positive co-occurrence of *Bifidobacterium* with *Faecalibacterium* in the EPS− vessel (*p* = 0.033; Figure S6). To determine putative EPS cross-feeding partners, we also analysed for co-occurrences with *B. breve* (i.e. specific for EPS+/EPS− supplemented strains), finding that, although not significant, there was a trend for more positive correlations with several genera in the EPS+ vessel including *Streptococcus* and *Ruminococcus* with *B. breve.* In general, we observed fewer positive correlations between EPS− and *B. breve*, with the genus *Dorea* significantly negatively correlated (Figure S6). Collectively, this might suggest that the presence of EPS is responsible for driving changes in microbiome profiles, likely due to the fact it can function as a potential metabolite for other members in the microbiota. 

### 3.3. Metabolite Profiles Shift in Response to Bifidobacterial EPS

**Figure 4:**
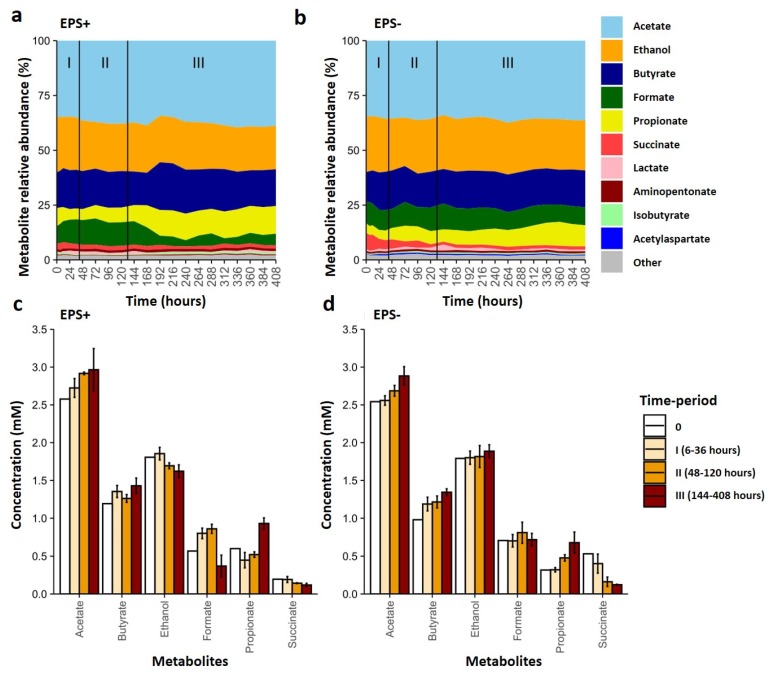
Metabolites profiling of EPS+ and EPS− vessels over time. Metabolite proportions as measured via 1H NMR, detected in (**a**) EPS+ and (**b**) EPS− vessels of model colon experiment calculated to 100%. Individual growth phases are denoted on each graph. The mean concentration of the most abundant (nM) metabolites within each time period (at 0 h, 6–36 h, 48–120 h and 120–408 h) for each vessel given either (**c**) *B. breve* UCC2003 EPS+ and (**d**) *B. breve* UCC2003del EPS−. Bars represent mean concentration for each metabolite over the specified time period, error bars represent standard deviation.

Using ^1^H NMR, we next sought to examine if the taxonomic compositional changes observed were also linked to overall metabolic changes. In agreement with the microbiota profiles, both vessels had similar starting metabolite concentrations prior to the introduction of *B. breve* (Figure S7a). The most abundant metabolites in each vessel were formate, succinate, ethanol, and bacterial fermentation by-product SCFAs: acetate, butyrate and propionate (Figure 4a–d; Table S4). Proportions of all SCFAs increased over time independent of the presence/absence of EPS (Figure 4c,d) [33]. We also observed that within the EPS− vessel, the concentration of ethanol was similar at the start and end, but with variations over time, including a peak from 120–168 h, whilst the EPS+ vessel appeared to have decreased levels. The abundance of the simple organic compound formate increased in abundance until 120 h within the EPS+ vessel, then dropped in the third phase (144–406 h) but remained stable in the EPS− vessel. We observed that the sugar compound succinate decreased with time in the EPS− vessel, whilst levels were relatively unchanged in the EPS+ system. Other notable differences in the EPS+ vessel included an increase in propionate during phase III (after 168 h). Taken together, these results further suggest that addition of bacteria with an EPS structure alters bacterial function and metabolic output. 

**Figure 5:**
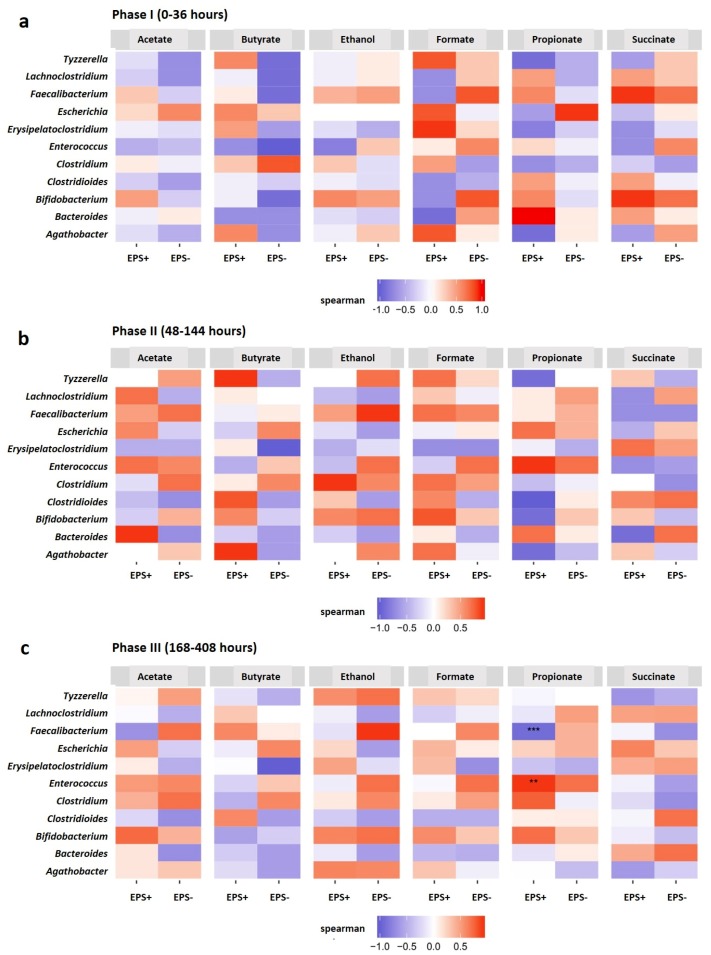
Spearman correlation between the top ten bacterial genera and top six abundant metabolites in each vessel (EPS+ vs. EPS−) at each phase; (**a**); phase I, (**b**); phase II, (**c**); phase III. *p < 0.05; **p < 0.01; ***p < 0.001. The *p*-values are adjusted for multiple comparison using Benjamini & Hochberg by genus, and by each vessel (EPS+ and EPS−).

Direct comparison of metabolic and microbiota profiles using Spearman correlation analysis identified changes, across phases and overall, that may be linked to EPS-mediated metabolic changes (Figure 2a, Figure 5 and Figure S7b,c). Overall, we noted more significant associations in the EPS+ vessel, and therefore chose to focus on the most abundant metabolites with the top ten bacterial genera across the experimental period (Figure S7b). The strongest positive correlations across the whole experimental period (i.e. both genus and metabolite increasing; *p* ≤ 0.001) were observed in the EPS+ vessel between *Bifidobacterium* and ethanol; *Tyzzerella, Escherichia, Clostridium, Agathobacter* and formate; *Enterococcus, Bacteroides* and propionate and, *Escherichia* and succinate (Figure S7b,c; Table S5). The presence of EPS+ *B. breve* was positively associated the SCFA butyrate and *Clostridioides* (and negatively associated in the EPS− vessel). Additionally, propionate was significantly negatively correlated with the proportion of *Tyzzerella* and *Agathobacter* in the EPS+ vessel and *Clostridioides* in the EPS− vessel. We also explored changes occurring during the different phases to more closely explore direct vs. indirect EPS metabolism associations (Figure 5). When *B. breve* was present at high abundance (i.e. Phase I) we noted (weakly) positive associations with *Tyzzerella* and butyrate in the EPS+ vessel, but the opposite (negative) associations in the EPS− vessel; a negative association was also observed for acetate and *Bacteroides,* and *Clostridioides* and formate. In phase II, we observed very similar positive associations in the EPS+ vessel (as in phase I) across genus and metabolites, although in some cases the associations appeared less strong which may correlate with reductions of EPS as a cross-feeding substrate (via loss of *B. breve*). The final phase III analysis indicated overall reductions in positive associations and metabolites in EPS+ vessel, and a move towards more negative associations which may be indicative of a complete loss of EPS as a nutrient at these later time-points. We did observe several new associations: a positive association with *Faecalibacterium* and butyrate in the EPS+ vessel, and a significantly strong negative association between *Faecalibacterium* and propionate, with the opposite (positive) trend with *Enterococcus* and propionate. The apparent differences in the metabolome with bacterial genus correlations indicate distinct and related changes due to the presence (or absence) of EPS, and may possibly infer active EPS metabolism (when present) by members of the wider early life microbiota.

## 4. Discussion

Previous studies have indicated that bifidobacterial EPS plays a key role in mediating beneficial microbe–host interactions [1]. Earlier studies have also suggested that EPS potentially acts as a dietary component, facilitating cross-feeding within microbial communities [20,21]. EPS molecules are large surface-bound polymers surrounding the cell wall of many Gram-positive bacteria, including *Bifidobacterium* species [12]. Expression of EPS may influence overall growth of *B. breve* due to increased energy expenditure, and/or EPS modulation of bacterial gene expression, which may impact profiles within complex microbial ecosystems [17]. RNASeq analysis indicated regulation of the EPS biosynthetic gene cluster (including glycosyltransferases), suggesting that EPS may act to modulate its own production, as indicated by TEM images [16]. Indeed, previous work in *Bacillus subtilis* indicates that the presence of EPS promotes the phosphorylation of a glycosyltransferase in the biosynthetic pathway, thereby stimulating the production of EPS [31]. We found that *in vitro*, presence of EPS did not significantly alter overall gene expression or growth, indicating any changes observed from the addition of *B. breve* UCC2003 EPS-positive/negative to a complex microbiota are due to presence (or absence) of EPS, rather than inherent cell response differences due to the additional exopolysaccharide motif.

Significant variations are often observed between infant microbiotas, particularly during weaning when ecosystems are undergoing rapid change in response to a changing nutritional environment (between 6–12 months of age). Our 16S rRNA amplicon analyses of the microbial ‘baseline’ model colon profiles revealed an expected weaning infant core microbiome; namely, dominance of the *Bacteroides* genus, and members of the phylum *Firmicutes (e.g., Clostridium* and *Erysipelatoclostridium),* with reduced abundance of *Bifidobacterium* [2,4,11]. This developmental window represents an important dietary transition period for infants, moving from milk (breast or formula) to solid food; therefore, access to additional microbial-derived nutrients, such as EPS, may contribute to driving ecosystem re-structuring [3]. Indeed, our research indicates that *B. breve* UCC2003 can modulate the infant microbial community structure and metabolism, and in line with previous studies we noted alterations in microbially-derived SCFAs, acetate, propionate and butyrate, although we did not detect the previously reported EPS driven acetate to propionate ratio change [20,21], which may be due to experimental and inoculating infant microbiota differences. However, as this is an individual model colon study the microbiota and the metabolite profiles presented only suggest putative direct and indirect EPS-associated modulations, careful interpretation of the data presented is required.

Previous studies on *B. breve* EPS has identified glucose and galactose as main components of EPS, which is similar to that proposed for UCC2003 EPS structure (includes glucose, galactose and/or the N-acetylated versions of these two sugars in different ratios or composition) [12,14,15,16]. The large molecular weight and structure of these EPSs may prevent host-associated digestion, allowing them to act as prebiotics within the colon (similar to inulin and fructooligosaccharide) [18,33,34]. From our analysis, we did not observe major shifts in the dominant microbiota genus during phase I in the EPS+ vessel, although at the mid-point in phase I and into phase II we noted an increase in the infant-gut associated bacteria *Tyzzerella* [4]. The timing of this increase suggests this early-life member may be using EPS as a key nutritional supplement. Although little is known regarding the metabolism of members the *Tyzzerella* genus, many other members of the family *Lachnospiraceae* possess glycoside hydrolases used for the metabolism and transport of oligosaccharides into the cell [35]. Furthermore, another member of the *Lachnospiraceae* family, *Ruminococcus*, degrades intestinal mucins (similar in structure to EPS) liberating metabolites for other members of the microbiota to consume [36]. Indeed, we also noted a positive association between *B. breve* and *Ruminococcus* (which was one of the lower relative abundance microbiota members), also in the EPS+ vessel. Thus, the positive correlation of *Tyzzerella* with formate (and butyrate) in the EPS+ vessels suggests that, similar to other members in the *Lachnospiraceae* family, *Tyzzerella* (and *Ruminococcus*) may be consuming EPS and producing SCFAs as fermentation end-products [37]. Moreover, we observed several other strong negative correlations between different genera (e.g., *Dorea*) and *B. breve* UCC2003, and *E. coli* only ‘blooming’ in the EPS− vessel in phase I, which also suggests antagonistic networks may be EPS-dependent, but further studies are required to understand the mechanisms promoting these inverse relationships. It should be noted that we only observed short-term ‘colonization’ of the respective *B. breve* strains; therefore, the earlier changes observed may link to direct metabolism, whilst the long-term changes in our model gut system (e.g., phase III increases in *Faecalibacterium* and *Enterococcus* and concurrent changes in propionate in the EPS+ vessel) may result from those initial cross-feeding interactions modulating the wider microbiota over time. Further studies using other *Bifidobacterium* species and strains with different EPS could provide further resolution on the importance of EPS acting as a putative dietary substrate in the wider microbiota over longer time-periods. Moreover, as this was a model colon study, with tight kinetic sampling, we did not perform multiple replicates (approach in line with other studies [38,39,40]), which is a limitation of this study. However, we did allow for stabilization as pre previous group protocols, and included mixed infant-associated fecal sample as the initial batch to reduce inter-microbiota variability [27,38,39]; further studies could inoculate individual infant ecosystems and explore the impact of EPS metabolism and cross-feeding interactions within and between different infants.

As highlighted above, the infant microbiota represents a dynamic ecosystem, with complex feeding networks at play, which may also be acting in response to EPS metabolism. We noted several potential wider cross-feeding networks (rather than direct EPS digestion interactions) during different growth phases. Specifically, utilization of *B. breve* EPS by proposed primary degraders highlighted from the genus-metabolites association analysis in phase I, e.g., *Bacteroides*, producing acetate may also facilitate the later growth of other microbiota members such as weaning-associated *Faecalibacterium*, important butyrate producers, as these metabolites correlate respectively with these genera [38,39]. Another possible interaction is via predicted production of formate by *Tyzzerella,* that may be metabolized by *Faecalibacterium,* as has been described in an *in silico* biofilm model [41]. Delineating intricate and complex cross-feeding relationships is difficult and cannot be predicted from non-community cultures, as strains grow and behave differently when grown in co-culture compared to those grown individually [19,42]. However, direct co-culture studies could provide some indications of the ability for certain microbiota members to directly metabolize EPS, like has been done previously for other dietary components such as human milk oligosaccharides [8,43]. Further, more in-depth studies, possibly utilizing isotope labelling, would be required for targeted identification of metabolites impacting these important EPS-associated cross-feeding networks [44]. 

The microbiota-associated changes observed after potential EPS metabolism and cross-feeding interactions may also be expected to impact host responses. Previous studies have shown a key role for direct EPS host modulation (including for *B. breve* UCC2003) via signaling through epithelial and immune cells [12,45]; however, changes in the wider microbiota via dietary interventions are also known to impact host responses [46]. Further studies using *in vivo* models, including gnotobiotic mice, or indeed human supplementation studies could provide insights into additional beneficial impacts of EPS-host interactions. 

## 5. Conclusions

To our knowledge, this research demonstrates for the first time the influence of *B. breve* UCC2003 EPS on the infant microbiota using an *in vitro* model colon system. Although a dynamic system, with media constantly replenished and waste removed, we observed short-term colonization that contributed to altered bacterial composition (i.e. abundance of *Tyzzerella*) and metabolite profiles (i.e. acetate, propionate, formate or butyrate) within the EPS-positive *B. breve* UCC2003 inoculated vessel. These combined data suggest EPS may act as a nutrient source for certain microbiota members, thereby driving community re-structuring during early life changes, although outputs should be interpreted carefully due to experimental limitations. Our results, in combination with previously published work [20,21], suggest that early life supplementation with EPS-positive *Bifidobacterium* strains may promote infant well-being by aiding and supporting beneficial infant microbiota development. Finally, this work, including the identification of microbial associations and metabolites within a healthy microbiota, could be used as a platform for development strategies of future nutritional and microbial therapies.

## Supplementary Materials

The following are available online at https://www.mdpi.com/2072-6643/12/4/948/s1, Figure S1: EPS-associated gene expression, Figure S2 Microbiota profiles for starting material used to inoculate EPS+ and EPS− vessels, Figure S3: Microbial changes over time as depicted using a NMDS plot using a Bray-Curtis dissimilarity calculation, Figure S4: 16S rRNA reads from each vessel EPS+ and EPS– that correspond to the total abundancy of *Bifidobacterium* reads, Figure S5: 16S rRNA genus bubble plot from the EPS+ and EPS− vessels in each phase, Figure S6: Correlation of either *Bifidobacterium* or *B. breve* with other genera in EPS+ and EPS− vessels, Figure S7: 1H NMR metabolic at t = 0 for EPS+ and EPS– vessels and Spearman correlation between top 10 and top 20 bacteria genera against all identified metabolites by ^1^H NMR, Table S1: Primer sequences for amplifying V1 + V2 region of 16S rRNA gene using MiSeq Illumina, Table S2: 16S rRNA genus raw reads for the EPS+ vessel, Table S3: 16S rRNA genus raw reads for the EPS− vessel, Table S4: Raw metabolite concentrations (in mM) for single metabolites for the EPS+ and EPS− vessel for each time point as measured by ^1^H-NMR, Table S5: Spearman correlation between 16S reads and all identified metabolite in each vessel (EPS+ vs. EPS−).
