# Supplementary material for: Bifidobacterium breve UCC2003 Exopolysaccharide Modulates the Early Life Microbiota by Acting as a Potential Dietary Substrate
Source: Nutrients. 2020 Mar 29;12(4):948. doi: 10.3390/nu12040948 (PMC7231044; doi:10.3390/nu12040948)
Supplement: Supplementary file 1 [file nutrients-12-00948-s001.zip › nutrients-704825-suppl-figure.pdf]

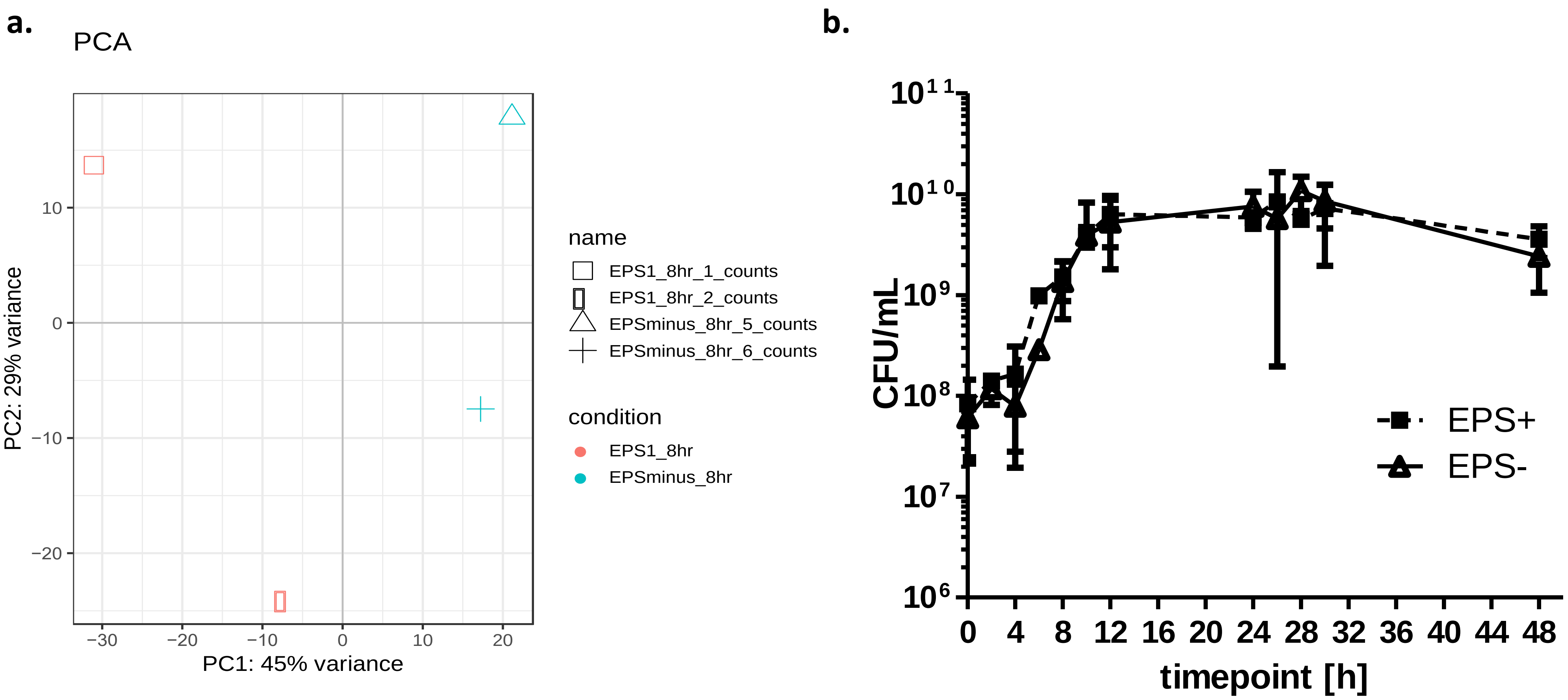

Supplementary Figure 1: EPS-associated gene expression. (a) Principle component analysis plot of RNASeq data from EPS+ and EPS- grown in rich media. Plots generated using normalised and regularised log transformed gene counts. **(b)** Growth kinetics of EPS+ and EPS- in MRS media plated in duplicate, error bars are ± standard deviation from three independent experiments.

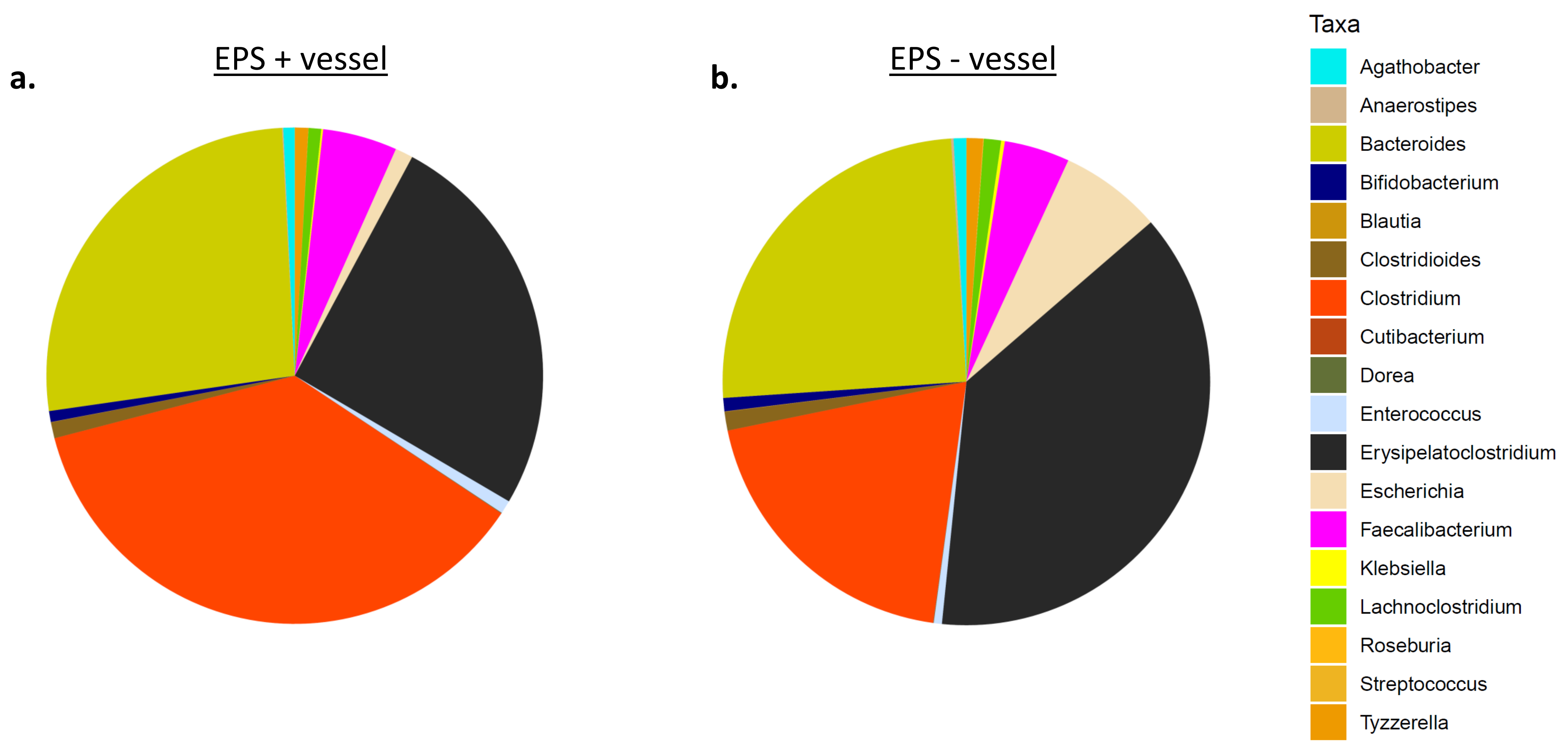

Supplementary Figure 2: Microbiota profiles for starting material used to inoculate **(a)** EPS+ and **(b)** EPS- vessels.

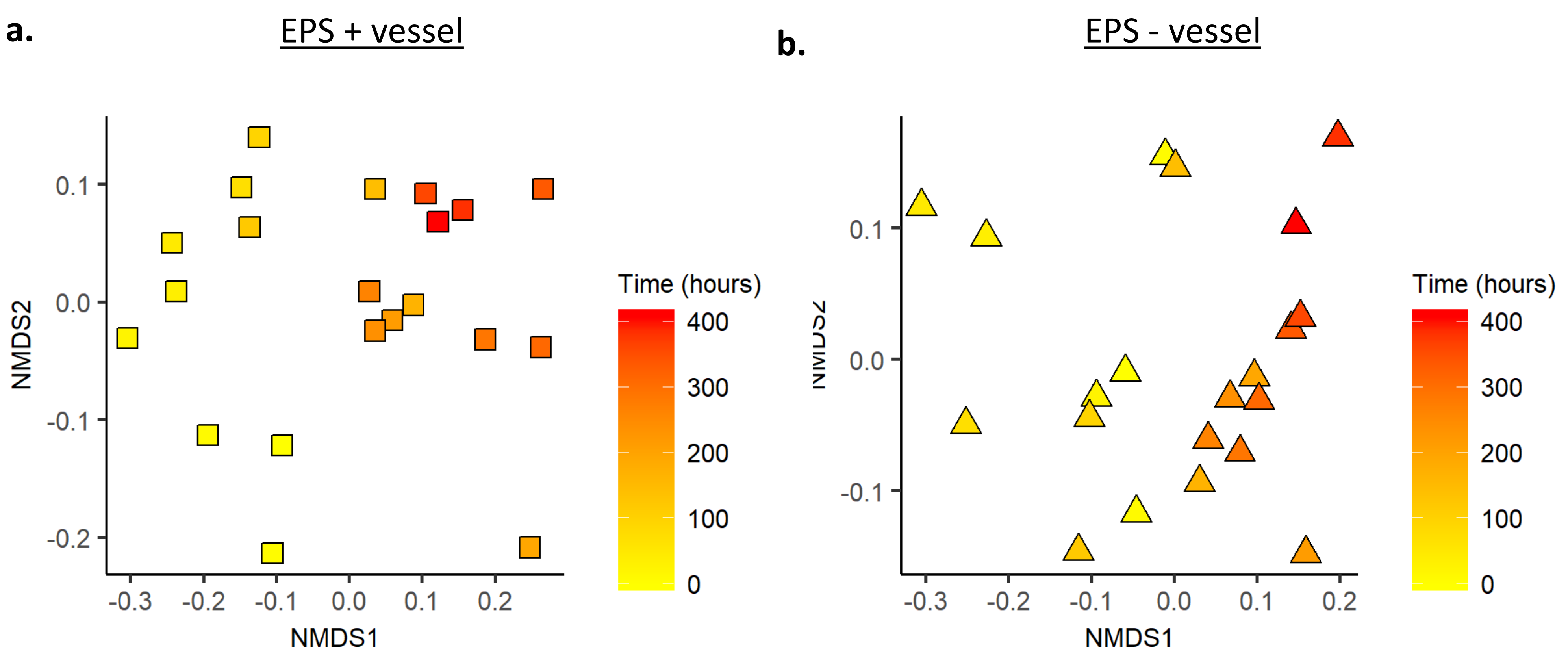

Supplementary Figure 3: Microbial changes over time as depicted using a NMDS plot using a Bray-Curtis dissimilarity calculation for **(a)** EPS+ □ and **(b)** EPS- △ vessels. Colour change denotes change in time from t=0 (yellow) to t=408h (red).

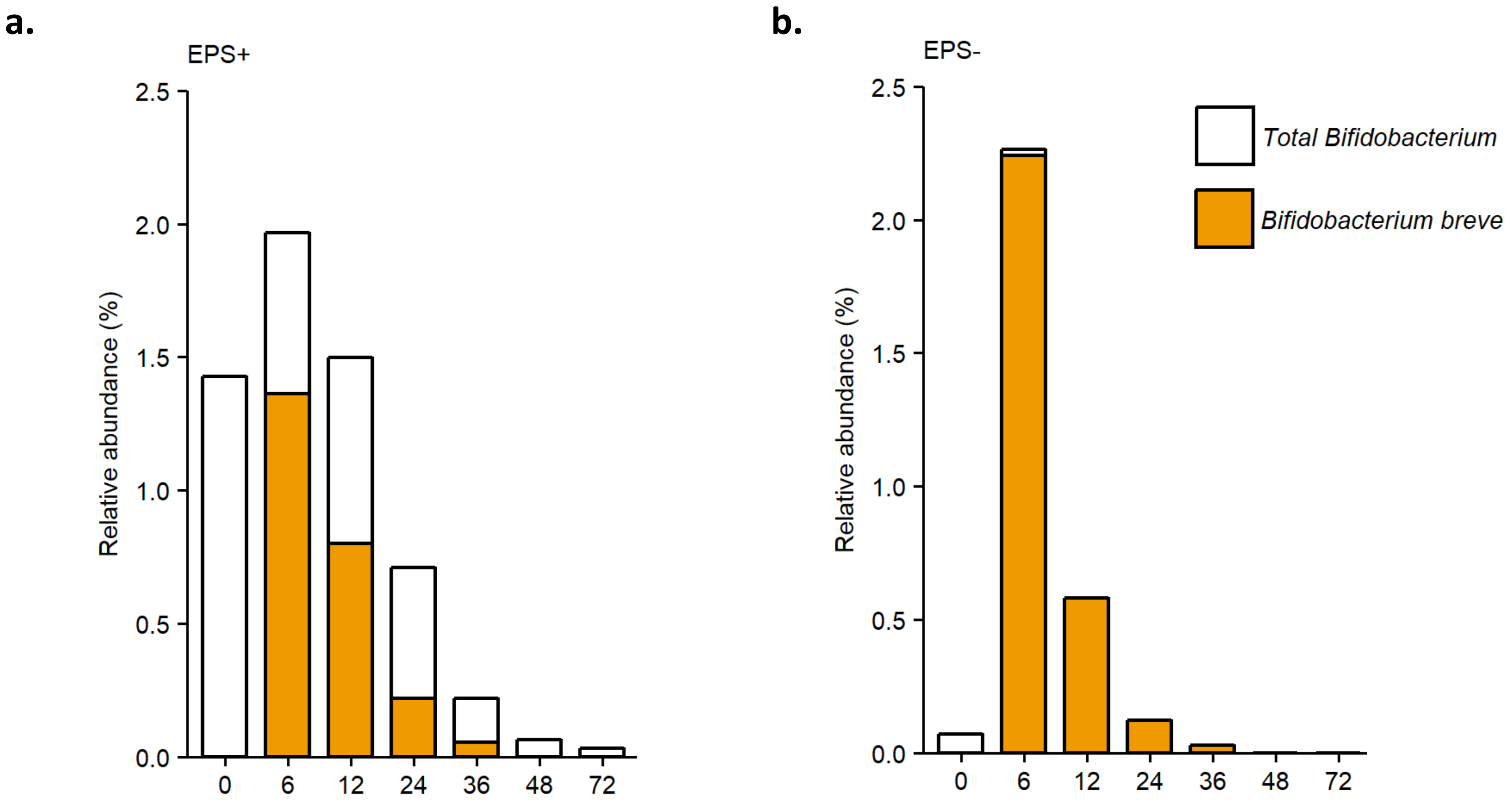

Supplementary Figure 4: 16S rRNA reads from each vessel **(a)** EPS + and **(b)** EPS – that correspond to the total abundance of *Bifidobacterium* reads in each vessels (white bars) and *B. breve* proportion (orange bars) over 72h. Note time point 0 correlates to the abundance of *Bifidobacterium* reads prior to inoculation with *B. breve* strains.

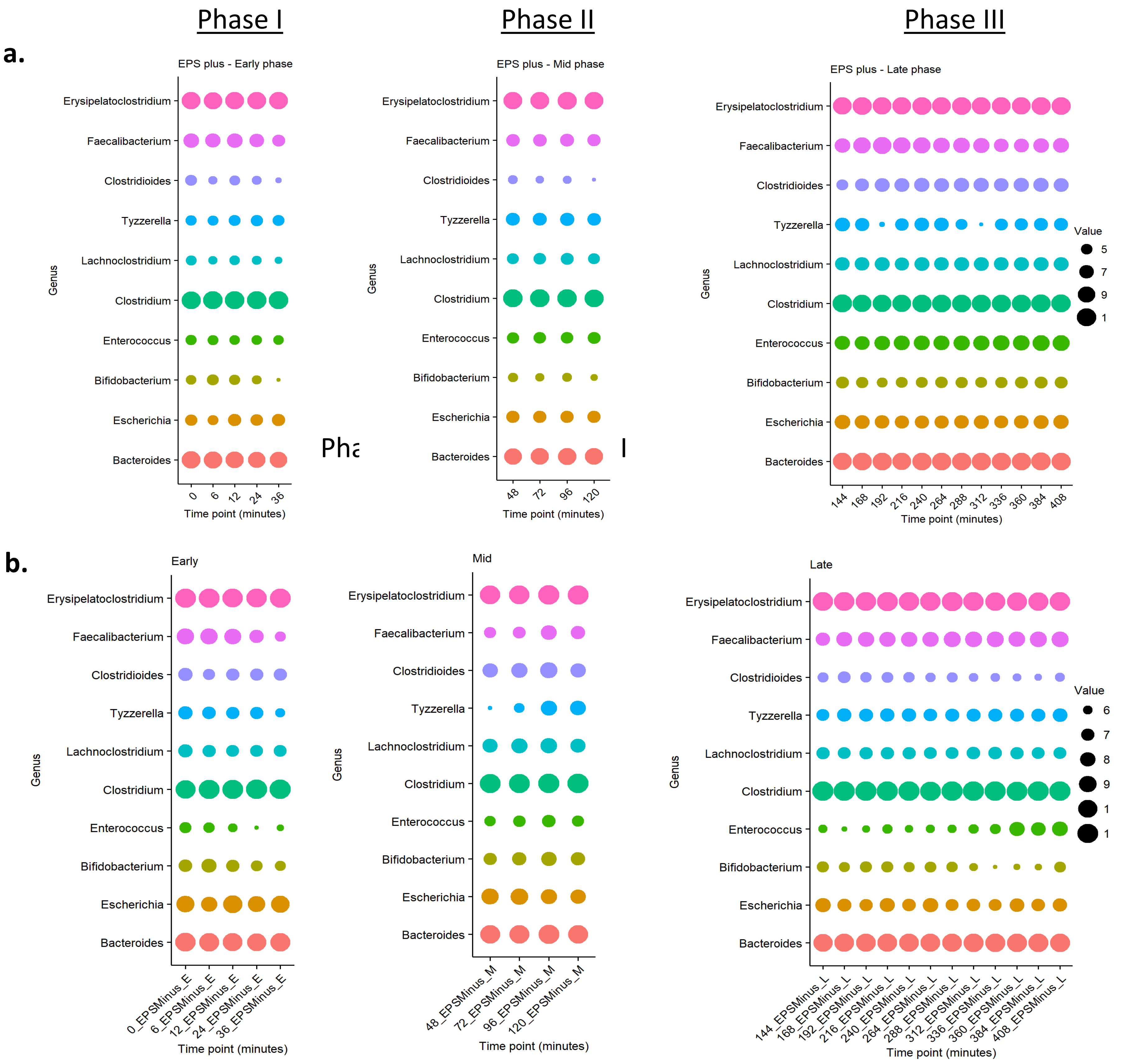

Supplementary Figure 5: 16S rRNA genus bubble plot from the (a) EPS+ and (b) EPS- vessels in each phase. Read counts were transferred into reference values to identify small abundance taxa. Samples are clustered on genera abundance.

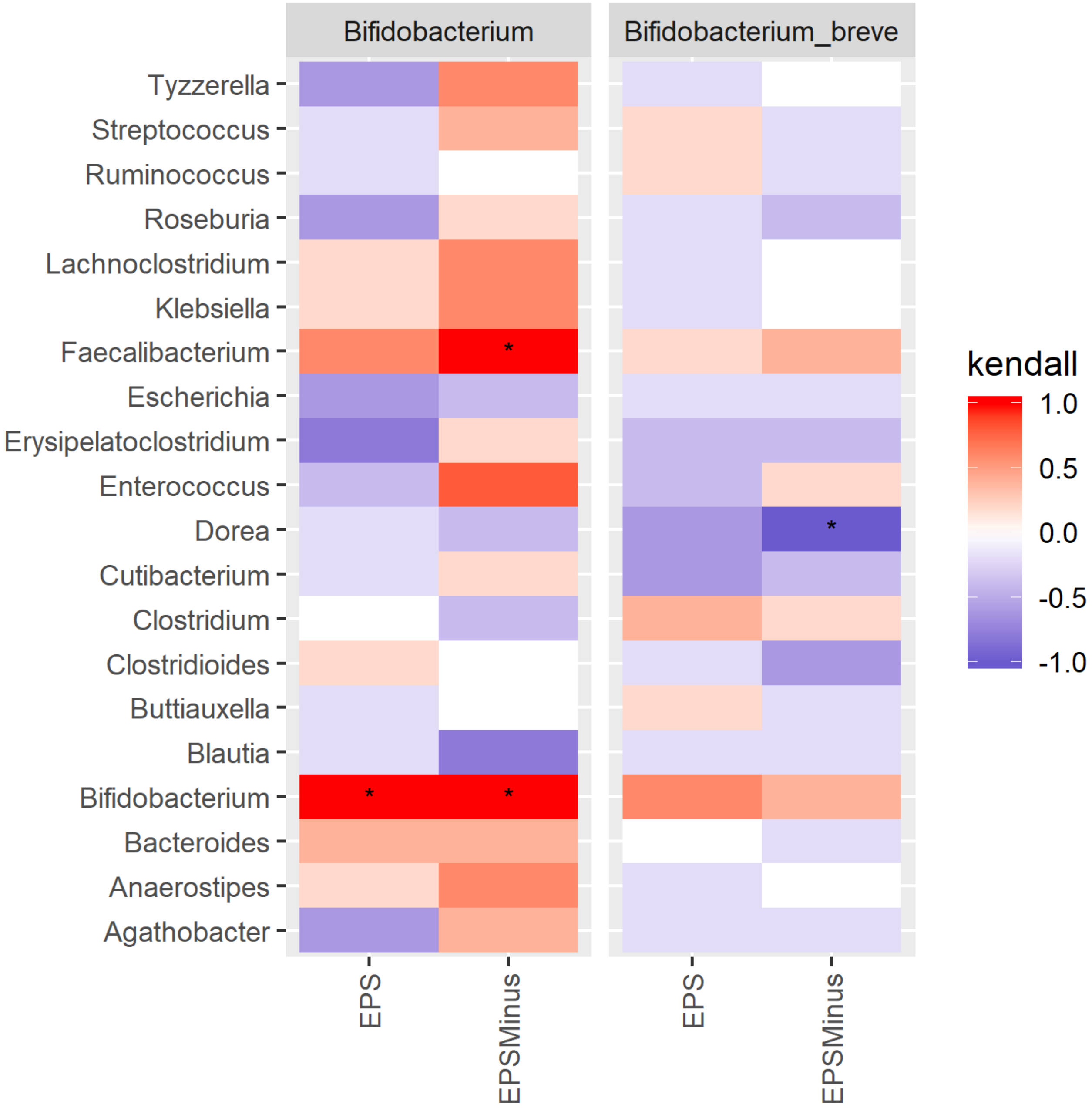

Supplementary Figure 6: Spearman correlation of either *Bifidobacterium* or *B. breve* with other genera in EPS+ and EPS- vessels (t=1-36h) using a Kendall rank correlation coefficient analysis in R Studio (*p=0.033).

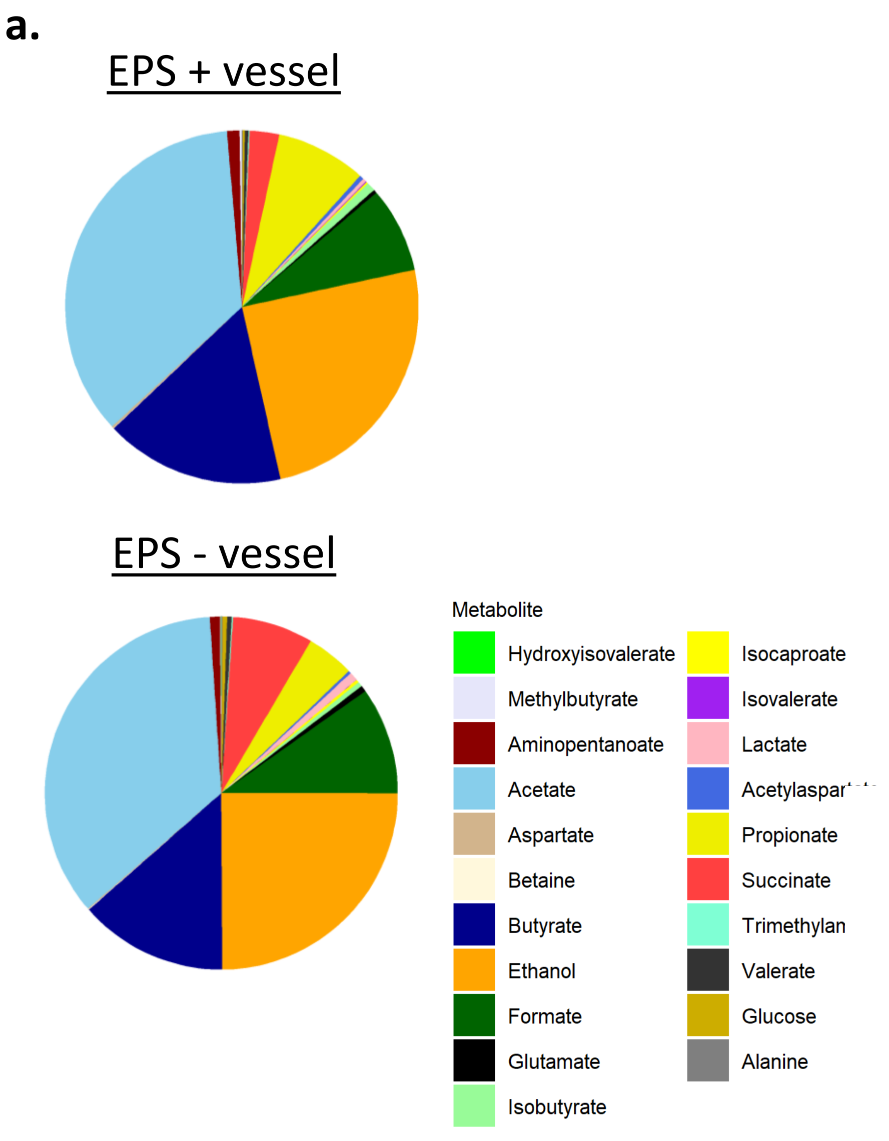

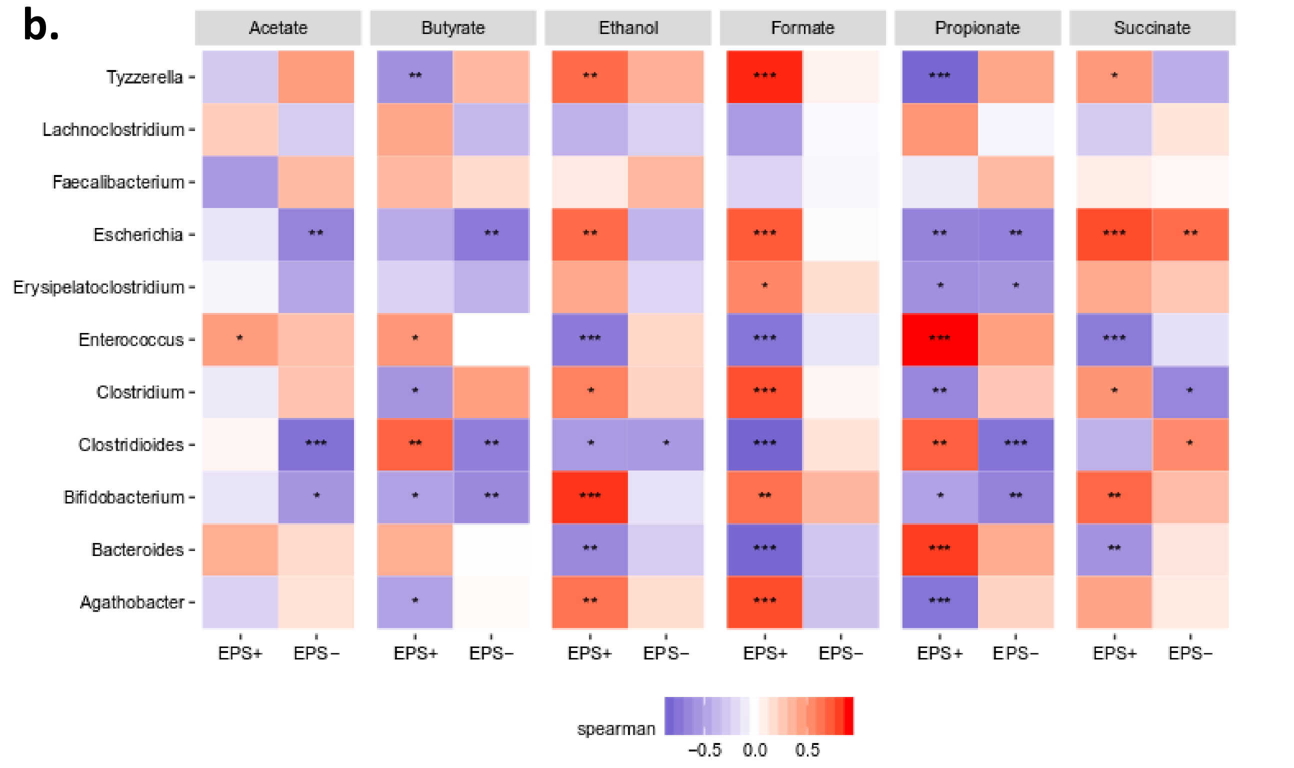

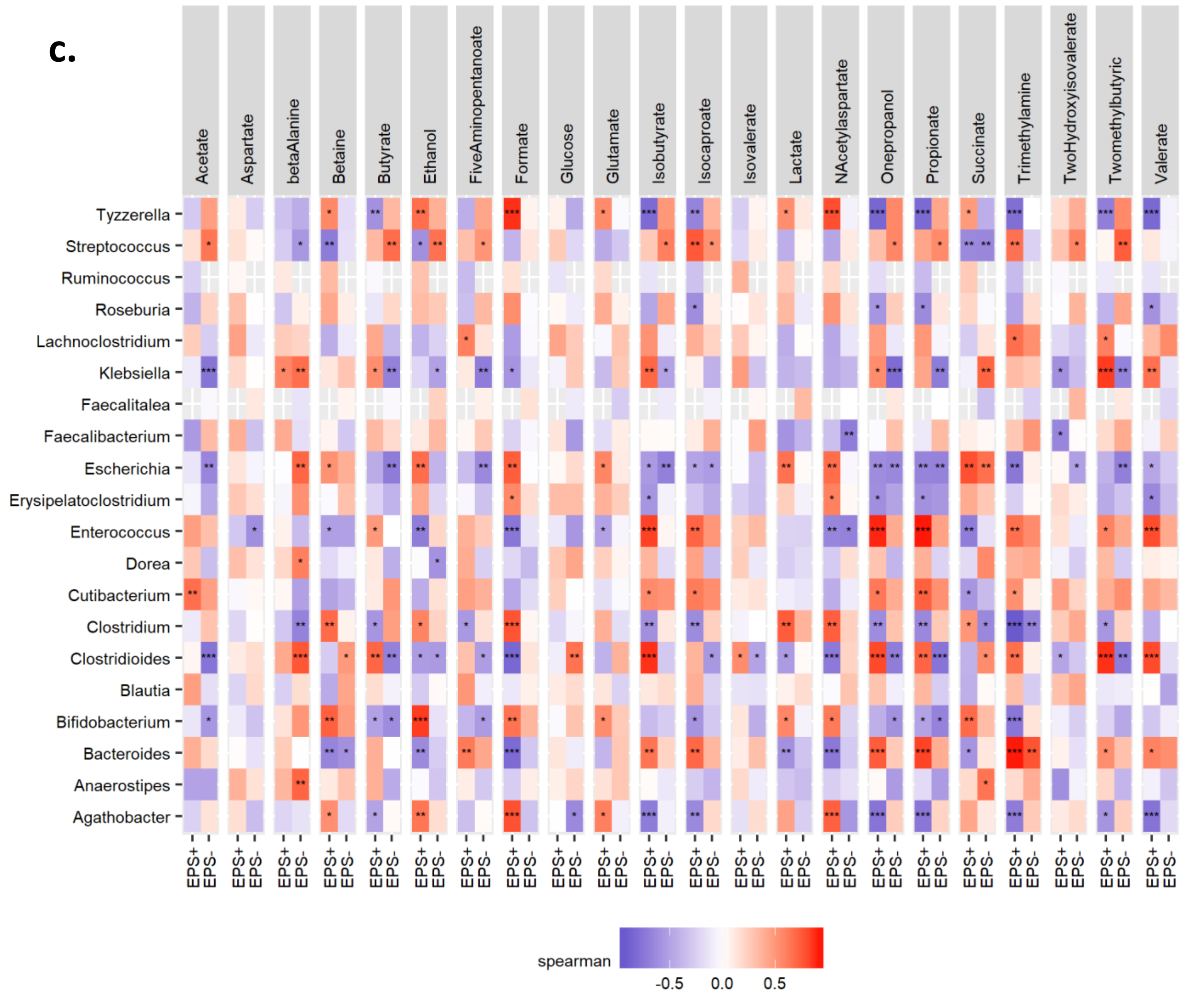

Supplementary Figure 7: (a) ^{1}H NMR metabolic at t=0 for EPS+ and EPS- vessels. (**b**) Spearman correlation between top 10 bacteria genera against all identified metabolites by ^{1}H NMR. (**c**) Spearman correlation between top 20 bacteria genera against all identified metabolites by ^{1}H NMR. *p < 0.05; **p<0.01; ***p<0.001; + indicates metabolite concentrations below detectable limits.
